# Ultrasound-derived gestational sac triple product as a predictor of early medical abortion failure with mifepristone-misoprostol regimens: a retrospective cohort study

**DOI:** 10.3389/fmed.2026.1780064

**Published:** 2026-04-07

**Authors:** Rui-Hong Xue, Juan Li, Yun-Xia Li, Zhao-Ying Gu, Dan Tang, Jing Yang, Yan-Yan Fu, Xin-Liang Chen, Ping Liu

**Affiliations:** 1 International Peace Maternity and Child Health Hospital, School of Medicine, Shanghai Jiao Tong University, Shanghai, China; 2 Shanghai Key Laboratory of Embryo Original Diseases, Shanghai, China; 3 Institute of Birth Defects and Rare Diseases, School of Medicine, Shanghai Jiao Tong University, Shanghai, China; 4 Karamay Central Hospital, Xinjiang, China

**Keywords:** early pregnancy, failure predictor, gestational sac size, medical abortion, ultrasound

## Abstract

**Objective:**

Early medical abortion (EMA) with mifepristone-misoprostol commonly relies on subjective metrics (e.g., last menstrual period-derived gestational age) for failure risk assessment, which may introduce uncertainty. This retrospective study aimed to identify objective predictors of EMA failure and explore the utility of different gestational sac size metrics for clinical risk stratification.

**Study design:**

Early pregnancy outpatients who underwent medical abortion with the mifepristone-misoprostol regimen at Karamay Central Hospital (Xinjiang, China) from December 2024 to July 2025 were included in our study. EMA failure was defined as the need for surgical evacuation. Analyses included univariate assessment, logistic regression, and performance evaluation of gestational sac size metrics.

**Results:**

After exclusion, 159 women were included in the final analyses. The overall EMA success rate was 89.31% (142/159). Maternal age ≥35 years was associated with higher failure risk (OR = 2.82, 95% CI, 1.01–7.89, *p* = 0.048). Gestational sac size emerged as an important objective correlate of EMA failure; among five assessed metrics (ellipsoid volume, maximum diameter, mean diameter, sum of three diameters, triple product of diameters), the triple product of diameters showed the highest correlation with failure (*r* = 0.316) and a significant association with failure risk (OR per 1,000 mm^3^ increase = 1.08, 95% CI, 1.03–1.14, *p* = 0.003). The triple product model demonstrated good discrimination, with an area under the curve (AUC) of 0.78 (95% CI, 0.68–0.88). Calibration was adequate (Hosmer-Lemeshow *p* = 0.62), and internal validation using bootstrap resampling confirmed stable performance (optimism-corrected AUC 0.76, 95% CI, 0.65–0.86). Its optimal cutoff (3648) yielded sensitivity = 0.765, specificity = 0.688, and Youden Index = 0.453. Fetal heart activity, embryonic bud presence, and parity were not significantly linked to failure (all *p* > 0.05).

**Conclusion:**

Gestational sac size is an important objective indicator for predicting mifepristone-misoprostol EMA failure, and among various gestational sac size metrics, the triple product of diameters demonstrates the highest predictive value.

## 1 Introduction

Medical abortion and surgical abortion offer pregnant individuals options for terminating pregnancy in the first trimester, empowering women with choice regarding their reproductive decisions under certain circumstances (1, 2). Mifepristone-misoprostol regimens for first- and second-trimester medical abortion are widely adopted, given their well-established efficacy and safety (3–5). However, pregnant individuals remain concerned about early medical abortion (EMA) failure, and healthcare providers—wary of potential disputes from such failures—often overemphasize perceived high failure rates during consultations. This dual concern discourages eligible patients from choosing EMA, restricting its clinical use and acceptability.

Current EMA failure risk assessment mainly relies on subjective/indirect measures (LMP-derived gestational age, clinical symptoms): LMP-based gestational age is biased by irregular cycles, ovulation variations, and inaccurate recall (6), while symptoms like vaginal bleeding are non-specific and fail to quantify risk, often leading to over/underestimation of risk. In contrast, ultrasound-derived gestational sac size is an objective, reproducible metric reflecting embryonic development that addresses these flaws, yet relevant studies remain limited. As the time has arrived to consider uniform early first-trimester (< 8 weeks’ gestation) ultrasound screening of all patients (7) further research on abortion outcomes disaggregated by visualization of the gestational sac is warranted (8).

Heikinheimo et al. (9) identified five epidemiological and clinical risk factors associated with medical abortion failure (surgical evacuation performed). Meaidi et al. (10) developed and validated a risk assessment model for predicting early medical abortion failure (surgical intervention performed) based on gestational age, maternal age, previous deliveries, and induced abortion history; however, the model was deemed inefficient as indicated by an area under the curve (AUC) of 0.63, and most outcomes remain unpredictable. In 2024, Liu et al. (11) developed and validated a more comprehensive model with 3 additional clinical parameters. However, existing EMA failure risk assessment models lack gestational sac size as a variable, warranting further research and validation for clinical application.

To address these gaps, this retrospective cohort study focused on outpatients undergoing EMA, aiming to analyze factors associated with EMA failure and explore the predictive value of objective ultrasound indicators. By integrating reliable quantitative data on such indicators, we sought to provide evidence for optimizing EMA risk stratification and advancing holistic abortion care (12). This prediction model is intended for use by obstetricians and gynecologists in outpatient settings to stratify patients’ risk of EMA failure, thereby facilitating personalized counseling and informed decision-making.

## 2 Materials and methods

### 2.1 Data collection

In accordance with the Declaration of Helsinki, the study was approved by the Ethics Committee on Human Research at Karamay Central Hospital, Xinjiang, China (Approval No.: YL-2025-083), which waived the need for informed consent to participate. After obtaining ethical approval, a retrospective cohort study was conducted to identify the risk factors for early pregnancy medical abortion (EMA) failure. Karamay Central Hospital is a regional medical and critical care center for pregnant and postpartum women in northern Xinjiang, an affiliated teaching hospital of Xinjiang Second Medical College, with an annual delivery volume of approximately 1,500–2,000.

Outpatients who underwent EMA at the hospital between December 2024 and July 2025 were included if they met the following criteria: gestational age <12 weeks; intrauterine pregnancy confirmed by routine transvaginal ultrasound (visible gestational sac and yolk sac) as more sensitive than abdominal ultrasonography (13); ectopic pregnancy excluded; voluntary request for pregnancy termination; opting for outpatient EMA after being informed of the pros and cons of surgical vs. medical abortion; no contraindications (e.g., glaucoma, asthma, abnormal liver function, abnormal vaginal discharge).

All patients received a mifepristone-misoprostol regimen based on the WHO-recommended guidelines (14), with protocol adjustments aligned with the application specifications in Chinese clinical practice guidelines for medical abortion (15, 16). Specifically, the standardized regimen was administered as follows: 200 mg mifepristone was given orally in a split dose (50 mg twice daily for 2 consecutive days), followed by 600 μg misoprostol via the oral route 24–48 h later; if no bleeding occurred 3 h after misoprostol administration, an additional dose of 200 μg misoprostol was given orally. If no bleeding was observed after a further 2 h, one more dose of 200 μg oral misoprostol was administered.

After physician-confirmed gestational sac expulsion and minimal vaginal bleeding, patients were discharged for home observation and advised to return for a follow-up ultrasound in 10–14 days.

EMA failure was defined as the need for surgical evacuation to complete the abortion (17, 18). Surgical evacuation was performed exclusively in patients who met both clinical and ultrasound criteria, with indications including excessive vaginal bleeding (≥80 mL within 24 h), prolonged bleeding (>2 weeks), or persistent abdominal pain, and persistent intrauterine residual tissue that persisted on follow-up ultrasound accompanied by an unsatisfactory decline in follow-up hCG levels that failed conservative treatment (additional misoprostol doses or uterotonic agents).

For patients who failed to attend scheduled follow-up visits, telephone follow-up was conducted throughout the study period to confirm the final abortion outcome. During these telephone interviews, participants were asked whether they had sought medical care at another hospital or experienced relevant symptoms (e.g., heavy or persistent vaginal bleeding). In the absence of such events, the abortion was deemed a successful outcome. Thus, the determination of medical abortion failure was derived from two sources: the hospital information system (for patients who attended follow-up visits) and telephone follow-up interviews (for those who did not). As our hospital serves as a regional medical center, study participants exhibited good treatment compliance and a relatively high follow-up revisit rate.

Data were extracted from the hospital’s outpatient medical record system, including maternal age, date of last menstrual period, gravidity, parity, surgical history, number of previous induced abortions, ultrasound findings (gestational sac size, presence and size of embryonic bud), serum human chorionic gonadotropin (hCG) levels, dates of medication administration, and follow-up records (size of intrauterine residues on follow-up ultrasound, interval between medication administration and follow-up ultrasound, follow-up serum hCG levels, details of medical treatment, and need for further intervention). For patients who underwent surgical intervention for failed outpatient medical abortion (including outpatient curettage and inpatient hysteroscopy), additional data were collected from surgical records, including the type of surgery and detailed operative notes.

### 2.2 Ultrasound measurements

*Equipment*: All measurements were performed using the transvaginal ultrasound system (GE Voluson E10) equipped with a 5–9 MHz endocavity probe, which was calibrated annually in accordance with the manufacturer’s standards.

*Measurement planes*: Transvaginal ultrasound scanning was used to measure the three mutually perpendicular dimensions (i.e., a, b, c diameters) of the gestational sac (GS) in two specific scanning planes, following a strictly defined measurement sequence. The superoinferior and anteroposterior diameters of the GS were obtained in the sagittal plane, and the laterolateral diameter was measured in the transverse plane; the maximum diameter of the GS was recorded as the largest value among the three orthogonal diameters. All three diameters represent orthogonal dimensions of the GS. Measurements were consistently taken from the inner edge of the chorionic membrane of the GS (i.e., the distance between the inner walls of the GS) to avoid inclusion of decidual tissue or decidual echoes, thereby preventing decidual tissue from being incorporated into the measurements.

*Operator training*: All measurements were performed by two sonographers with at least 5 years of clinical experience in obstetric ultrasound, after completing standardized training on the GS measurement protocol.

*Repeats and averaging*: Each of the three diameters (superoinferior, anteroposterior, and laterolateral) was measured three consecutive times, and the mean value was used for subsequent statistical calculations. If the range of three repeated measurements for any diameter exceeded 2 mm, the measurement was repeated by the same operator. If discrepancies persisted after re-measurement, a final confirmatory measurement was performed by a senior sonographer with at least 10 years of experience.

*Timing of measurements*: In the absence of contraindications, all ultrasound measurements were completed within 24 h before mifepristone administration (baseline) to ensure consistency with the study’s exposure window (i.e., GS size at the initiation of medical abortion).

### 2.3 Data analysis

Prior to analysis, all data were checked for completeness and consistency. Outliers were examined by visualizing distributions, and data entry errors were corrected by referring to original medical records. Descriptive statistics were presented as medians with interquartile ranges (25th–75th percentiles) for continuous variables and frequencies with percentages for categorical variables.

**Figure 1:**
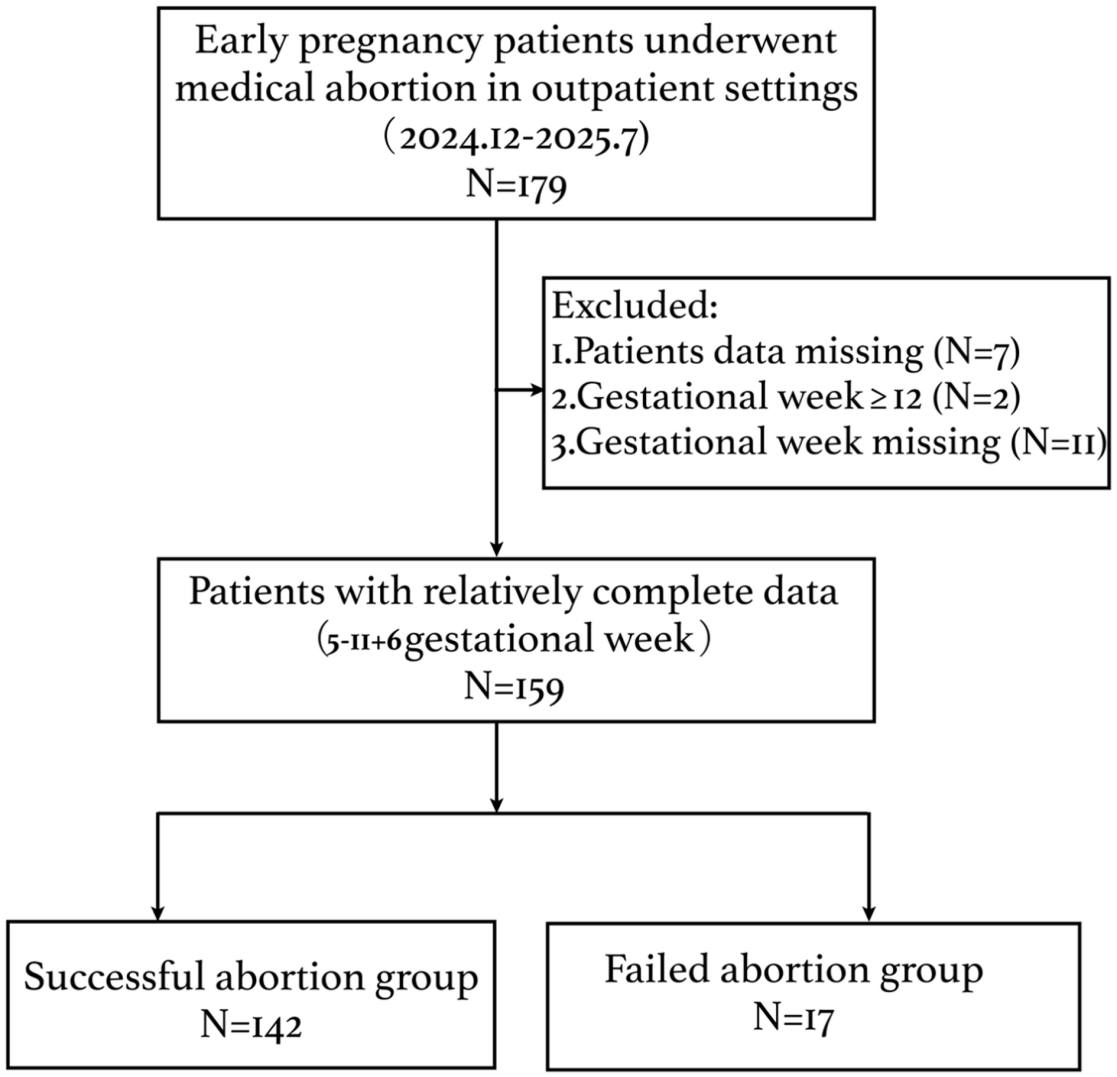
Flowchart of the study.

Due to the retrospective design, no formal a priori sample size calculation was performed. Instead, we assessed the adequacy of the sample size for logistic regression using the events per variable (EPV) criterion, which is a widely accepted method to evaluate overfitting risk. Post-hoc, with 159 participants and 17 outcome events, the EPV was 17 (17 events/1 predictor in the final model), exceeding the commonly recommended threshold of 10 and indicating adequate sample size for the univariate analysis. Internal validation using bootstrap resampling was nevertheless performed to confirm model stability. Missing data were handled by complete case analysis, as the proportion of missingness was low for most variables (except parity, which had 40.3% missing). Parity was excluded from multivariable modeling. Due to the high missing rate in parity, we performed missingness analysis and multiple imputation as sensitivity analyses. For the remaining variables, participants with any missing data (*n* = 7) were excluded from the analysis, as shown in Figure 1.

We employed multiple analytical approaches to examine the relationship between gestational sac measurements and medical abortion failure. The continuous predictor (triple product) was modeled as a linear term without transformation, based on visual inspection of its relationship with the logit of the outcome using lowess smoothing. No rescaling or standardization was applied to maintain interpretability of the original measurement units. Logistic regression analysis was used to identify risk factors for abortion failure, with results expressed as odds ratios (ORs) and 95% confidence intervals (CIs). Receiver operating-characteristic (ROC) curve analysis was conducted to determine the optimal cutoff values for gestational sac measurements in predicting abortion failure, with area under the curve (AUC) calculated to assess predictive accuracy.

For comprehensive visualization of multivariate relationships, we generated several graphical representations: probability scatter plots with fitted logistic curves displayed the continuous relationship between individual measurements and failure probability, while correlation heatmaps illustrated the strength and direction of associations between multiple gestational sac parameters (maximum diameter, triple product, ellipsoid volume, sum of three diameters, and mean diameter) and abortion outcomes. Failure rate heatmaps further delineated risk stratification across measurement quantiles. The triple product (a × b × c, where a, b, c are the three perpendicular diameters of the gestational sac) was selected based on three key considerations: scientific validity and rational basis, clinical feasibility, and unaddressed research gaps. The triple product integrates three-dimensional information, capturing the true volume-related growth of the gestational sac more accurately. The ellipsoid volume (V) is calculated as V = (*π*/6) × triple product, indicating a direct proportional relationship between the two metrics.

We assessed the predictive performance of the triple product model in terms of discrimination and calibration, followed by internal validation using bootstrap resampling.

Discrimination was quantified by the area under the receiver operating-characteristic curve (AUC) with its 95% confidence interval (CI) estimated by 1,000 bootstrap replicates.

Calibration was evaluated by three approaches: (i) calibration-in-the-large (intercept) and calibration slope, obtained by refitting a logistic regression model with the linear predictor as an offset and as a single covariate; (ii) the Hosmer-Lemeshow goodness-of-fit test, which compares observed and predicted event rates across deciles of predicted risk; and (iii) a calibration plot, where predicted probabilities were plotted against observed frequencies, smoothed by loess regression.

Internal validation was performed using bootstrap optimism correction. One thousand bootstrap samples were drawn with replacement from the original dataset. In each sample, the model was re-fitted and evaluated both on the bootstrap sample (apparent performance) and on the original data (test performance). The optimism was defined as the mean difference between the apparent and test AUC across bootstrap samples, and the optimism-corrected AUC was obtained by subtracting this optimism from the original AUC. The 95% CI of the corrected AUC was derived from the 2.5th and 97.5th percentiles of the bootstrap distribution.

Logistic analyses were performed using SPSS version 23.0 (IBM, USA). Other analyses were conducted using R software (version 4.5.1) with packages ‘pROC’, ‘rms’, and ‘boot’. Statistical analyses utilized the following R packages: ggplot 2 for data visualization, dplyr and tidyr for data manipulation, and ggpubr for statistical annotations. A two-sided *p*-value < 0.05 was considered statistically significant throughout all analyses.

## 3 Results

### 3.1 Study population characteristics

After retrospective review of the outpatients’ digital records between December 2024 and July 2025 at Karamay Central Hospital (Xinjiang, China), 179 outpatients who underwent medical abortion were included. Women with missing data (*n* = 7), gestational age ≥12 weeks (*n* = 2), or unknown gestational age (*n* = 11) were excluded, resulting in a final analytical sample of 159 patients with complete data (gestational age range: 5–11 + 6 weeks). Of these 159 patients, 142 (89.31%) achieved successful medical abortion, while 17 (10.69%) experienced failure requiring surgical evacuation (Figure 1).

Baseline clinical characteristics of the included patients are summarized in Table 1. The median maternal age was 30 years (interquartile range [IQR], 25–35 years), with a median parity (number of previous live births) of 1 (IQR, 0–1). The median interval from medication administration to follow-up ultrasound examination was 13 days (IQR, 11–17 days) (Table 1).

**Table 1:** Clinical characteristics of patients included in the study period.

Characteristics	Median (25th–75th percentile)
Maternal age (years)	30 (25, 35)
Gestational age (weeks)	6.6 (6.0, 7.6)
Parity(n)	1(0, 1)
Intervals to ultrasound (days)	13(11, 17)

Table shows the baseline clinical characteristics of the 159 included early-pregnancy outpatients (with complete data). Continuous variables (maternal age, gestational age, parity, ultrasound interval) are presented as median (IQR), providing an overview of the study population’s features to support subsequent analyses.

### 3.2 Univariate analysis of factors associated with medical abortion failure

Table 2 presents the univariate analysis of associations between clinical variables and medical abortion failure. Among the evaluated variables, maternal age and gestational sac size were significantly correlated with abortion failure (both *p* < 0.05), while other factors (parity, fetal heart beats, embryonic bud presence, embryonic bud size) showed no significant associations (all *p* > 0.05). Patients aged ≥35 years had a significantly higher abortion failure rate (19.05%, 8/42) compared to those aged <35 years (7.69%, 9/117). Using patients aged <35 years as the reference group, the odds ratio (OR) for failure in the ≥35 years group was 2.82 (95% confidence interval [CI], 1.01–7.89; *p* = 0.048). Patients with a gestational sac maximum diameter ≥30 mm had a substantially higher failure rate (33.33%, 7/21) than those with a diameter <30 mm (7.25%, 10/138). With the <30 mm group as reference, the OR for failure in the ≥30 mm group was 6.40 (95% CI, 2.10–19.47; *p* = 0.001), indicating a more than 6-fold increased risk of failure. However, no significant differences in medical abortion failure rates were found with respect to parity, fetal heartbeat status, presence of an embryonic bud, or embryonic bud size (all *p* > 0.05) (Table 2).

**Table 2:** Correlation between failure of medical abortion and different variables.

Variables	Total medical abortions		Failure of medical abortions			
	Total, n†	(%)	Failures, *n*	(%)^&^	Odds ratio (95% CI)	*P*
Total	159	(100)	17	(10.69)		
Maternal age (y)						
<35	117	(73.58)	9	(7.69)	1 (reference)	0.048
≥35	42	(26.42)	8	(19.05)	2.82 (1.01, 7.89)*	
Parity (n)						
<1	24	(15.10)	1	(4.17)	1 (reference)	0.218
≥1	71	(44.65)	10	(14.08)	3.77 (0.46, 31.13)	
Missing‡	64	(40.25)	6	(9.38)		
Fetal heart beats						
Not with	107	(67.30)	10	(9.35)	1 (reference)	0.433
With	52	(32.70)	7	(13.46)	1.51 (0.54, 4.22)	
Embryonic bud						
No bud detected	93	(58.49)	8	(8.60)	1 (reference)	0.315
Embryonic bud detected	66	(41.51)	9	(13.64)	1.68 (0.61, 4.61)	
Embryonic bud size						
<3 mm	111	(69.81)	11	(9.91)	1 (reference)	0.628
≥3 mm	48	(30.19)	6	(12.50)	1.30 (0.45, 3.74)	
Gestational sac size						
<30 mm	138	(86.79)	10	(7.25)	1 (reference)	0.001
≥30 mm	21	(13.21)	7	(33.33)	6.40 (2.10, 19.47)*	

Table presents univariate analysis results of 8 variables linked to medical abortion failure. Only maternal age and gestational sac size were significantly associated with failure (*P* < 0.05); the other variables (parity) were not (*P* > 0.05). Key indicators (failure rate, OR, 95% CI, *P* -value) are included for each variable. †This sum does not necessarily equal the sample size for all variables because some data were missing. ‡Parity data missing due to incomplete medical records. ^&^Failure rates are calculated as (number of failures/number of cases in group) × 100%, rounded to two decimal places. *P < 0.05 (compared with reference during every characteristic unit).

### 3.3 Sensitivity analysis of gestational sac size as an independent predictor

After adjusting for maternal age alone, for maternal age and gestational age, and for all five covariates (with multiple imputation for parity), gestational sac size ≥30 mm remained significantly associated with failure (all *p* < 0.05), confirming it as an independent risk factor (Table 3). Supplementary Table S1 indicates that most variables showed no significant differences. However, significant differences were observed for maternal age (*p* = 0.0003) and embryonic bud size (*p* = 0.0186). These differences indicate that the missingness of parity is associated with observed factors such as maternal age. This pattern supports the assumption that the data are missing at random (MAR) (Supplementary Table S1). Sensitivity analyses addressing missing parity data (40.3%) confirmed the robustness of our findings. After multiple imputation, gestational sac size remained the only significant predictor of abortion failure, with effect sizes consistent with the primary analysis. Complete case analysis and alternative imputation methods yielded virtually identical results (Supplementary Table S2). Multivariable analysis adjusting for all five covariates confirmed that gestational sac size remained the strongest independent predictor (adjusted OR = 10.02, *p* = 0.003; Supplementary Table S3, Supplementary Figure S1).

**Table 3:** Sensitivity analyses of correlations between gestational sac size and failure of abortion.

Risk factor						
Gestational sac size	AOR*	P	AOR^+^	P	AOR^#^	P
<30 mm	1 (reference)	0.002	1 (reference)	0.009	1 (reference) 10.02 (2.27–44.13)	0.003
≥30 mm	5.79 (1.87,17.94)		5.72 (1.55, 21.04)			
	*AOR adjusted for maternal age.		^+^AOR adjusted for maternal age, and gestational age.		^#^AOR adjusted for all five covariates after multiple imputation of parity	

Table shows sensitivity analysis results for gestational sac size (≥30 mm vs <30 mm) as a risk factor for abortion failure. After adjusting for maternal age alone, then maternal age and gestational age and all five covariates (maternal age, parity, embryonic bud, embryonic bud size, fetal heart beats), gestational sac size remained significantly associated with failure (*p* < 0.05), confirming it as an independent risk factor.

### 3.4 Association between gestational sac measurement indices and medical abortion failure, and predictive performance of cutoff values

To clarify the relationship between gestational sac size and EMA failure, and establish a practical predictive cutoff, we analyzed five indices: ellipsoid volume = (*π*/6) a × b × c, maximum diameter (maximum in a, b, and c), mean diameter[=(a + b + c)/3], sum of three diameters (=a + b + c), and triple product (=a × b × c), among which a, b, and c, respectively, represent the three dimensions (length, width, and thickness) of the gestational sac measured by ultrasound.

**Figure 2:**
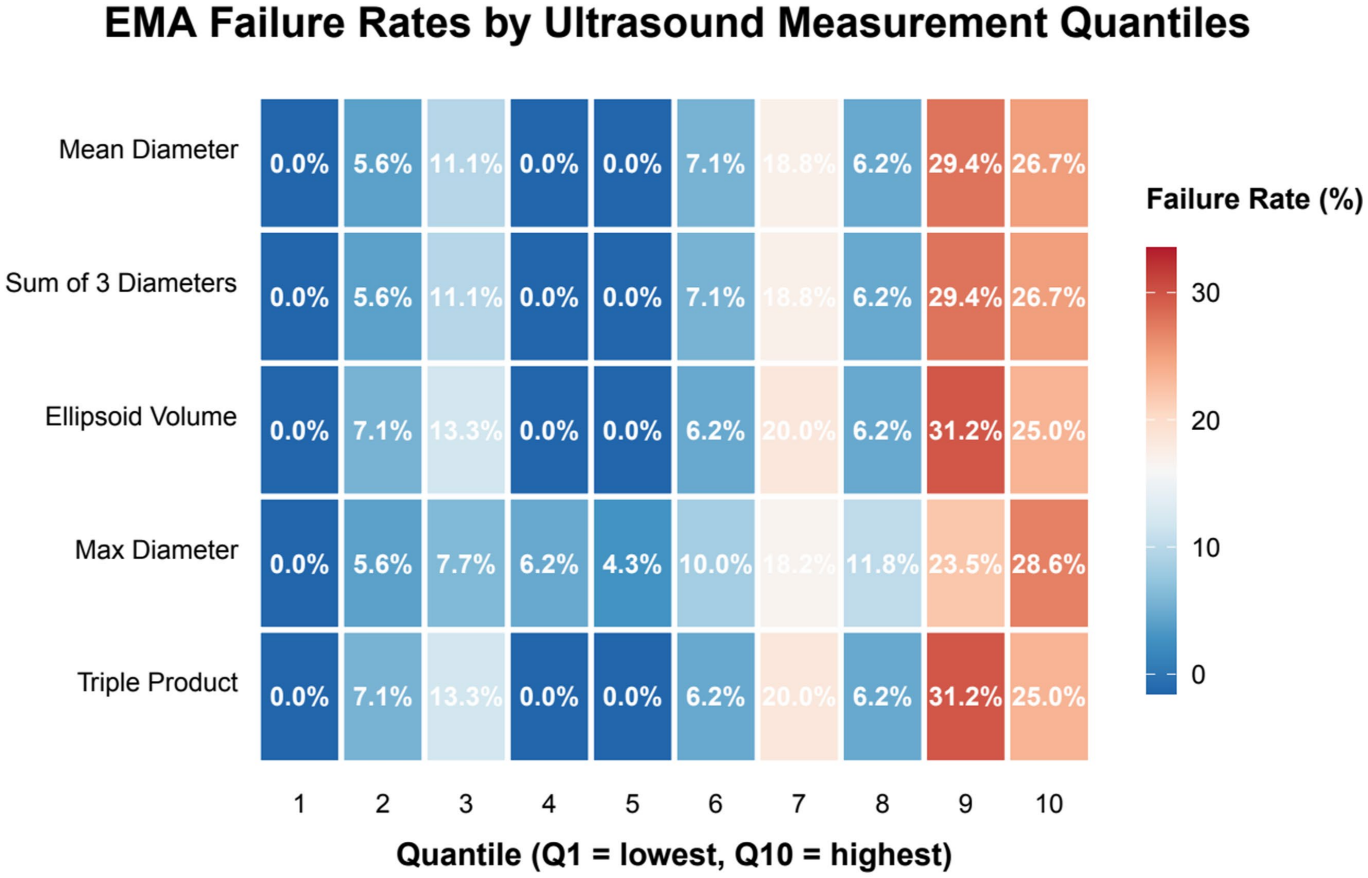
Association between gestational sac measurement metrics and EMA failure rate across quantiles. This heatmap illustrates the failure rate of EMA across ten quantiles (1 = lowest to 10 = highest) for five gestational sac measurement metrics: mean diameter, sum of three diameters, ellipsoid volume, maximum diameter, and triple product. The triple product and ellipsoid volume demonstrated the highest failure rates in the highest quantiles (31.2% in quantile 9 for triple product), suggesting these indices may have stronger predictive value for EMA failure compared to others.

**Figure 3:**
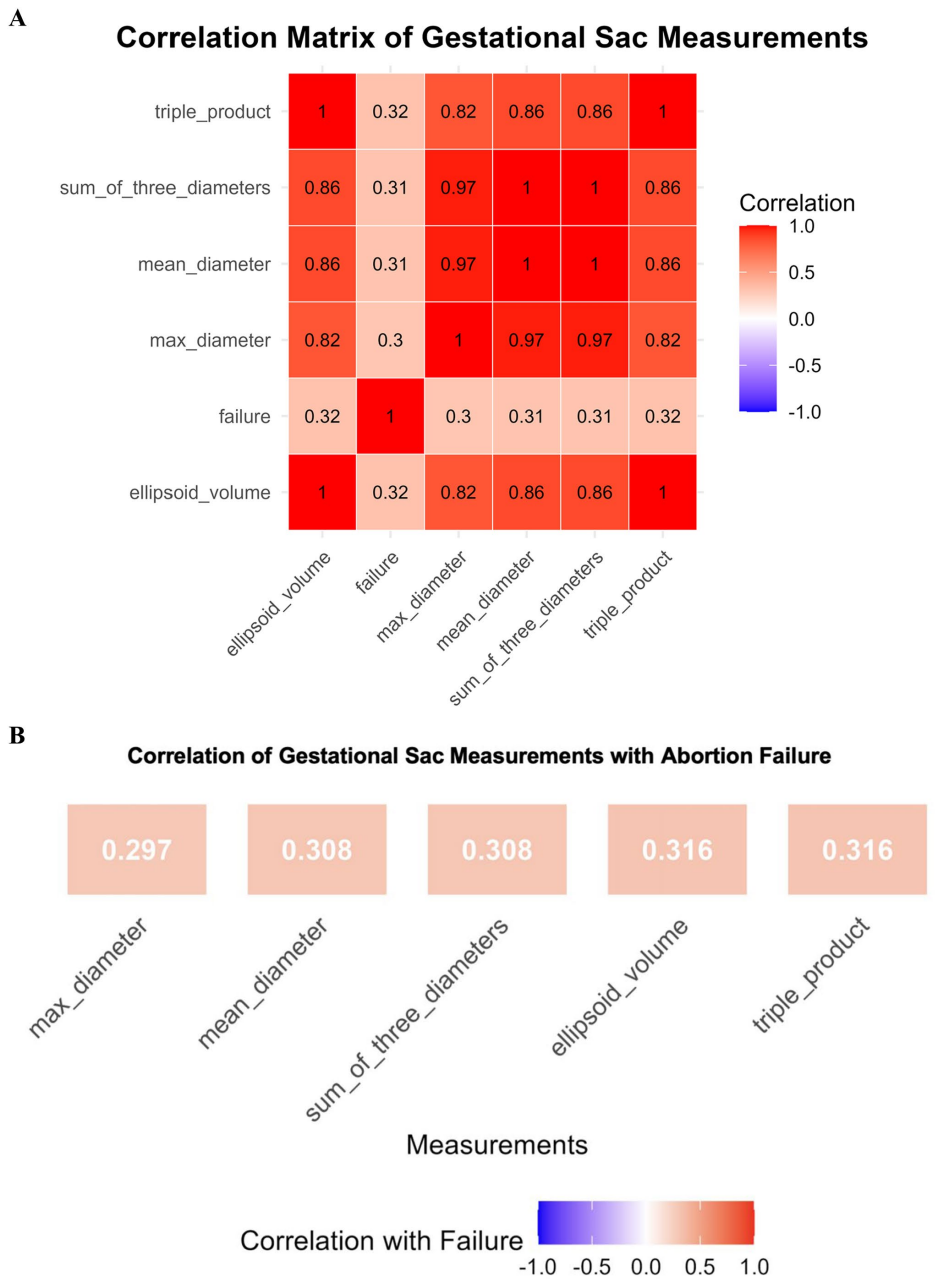
**(A)** Correlation matrix of gestational sac measurements. Heatmap displaying Pearson correlation coefficients among five gestational sac parameters: triple product, sum of three diameters, mean diameter, maximum diameter, and ellipsoid volume. Color intensity indicates the strength and direction of correlation, with red representing positive correlations and blue representing negative correlations. All dimensional measurements showed strong intercorrelations (*r* > 0.80), while correlations with EMA failure were weak (*r* ≈ 0.30–0.32). **(B)** Correlation of gestational sac measurements with abortion failure. Bar plot illustrating the Pearson correlation coefficients between individual gestational sac measurements and abortion failure outcome. Ellipsoid volume and triple product demonstrated the highest correlations with failure (*r* = 0.316), whereas maximum diameter showed the lowest (*r* = 0.297). The color scale indicates correlation strength, with warmer colors representing stronger positive associations.

The triple product and ellipsoid volume demonstrated the highest failure rates, reaching 31.2% in quantile 9 (Figure 2). Pearson correlation analysis was performed to examine the interrelationships among gestational sac measurements. As shown in Figure 3A, strong positive correlations were observed among all dimensional parameters, with correlation coefficients ranging from 0.82 to 1.00. Specifically, mean diameter, sum of three diameters, and maximum diameter exhibited very high intercorrelations (*r* ≥ 0.97), as did the volume-derived measures (ellipsoid volume and triple product, *r* = 1.00). Notably, all measurement parameters demonstrated weak positive correlations with EMA failure, with coefficients ranging from 0.30 to 0.32. To further quantify the predictive value of individual gestational sac measurements for EMA failure, we analyzed the correlation between each parameter and EMA failure (Figure 3B). Ellipsoid volume and triple product showed the strongest associations with abortion failure (*r* = 0.316 for both), followed by mean diameter and sum of three diameters (*r* = 0.308 for both), while maximum diameter demonstrated the weakest correlation (*r* = 0.297).

**Figure 4:**
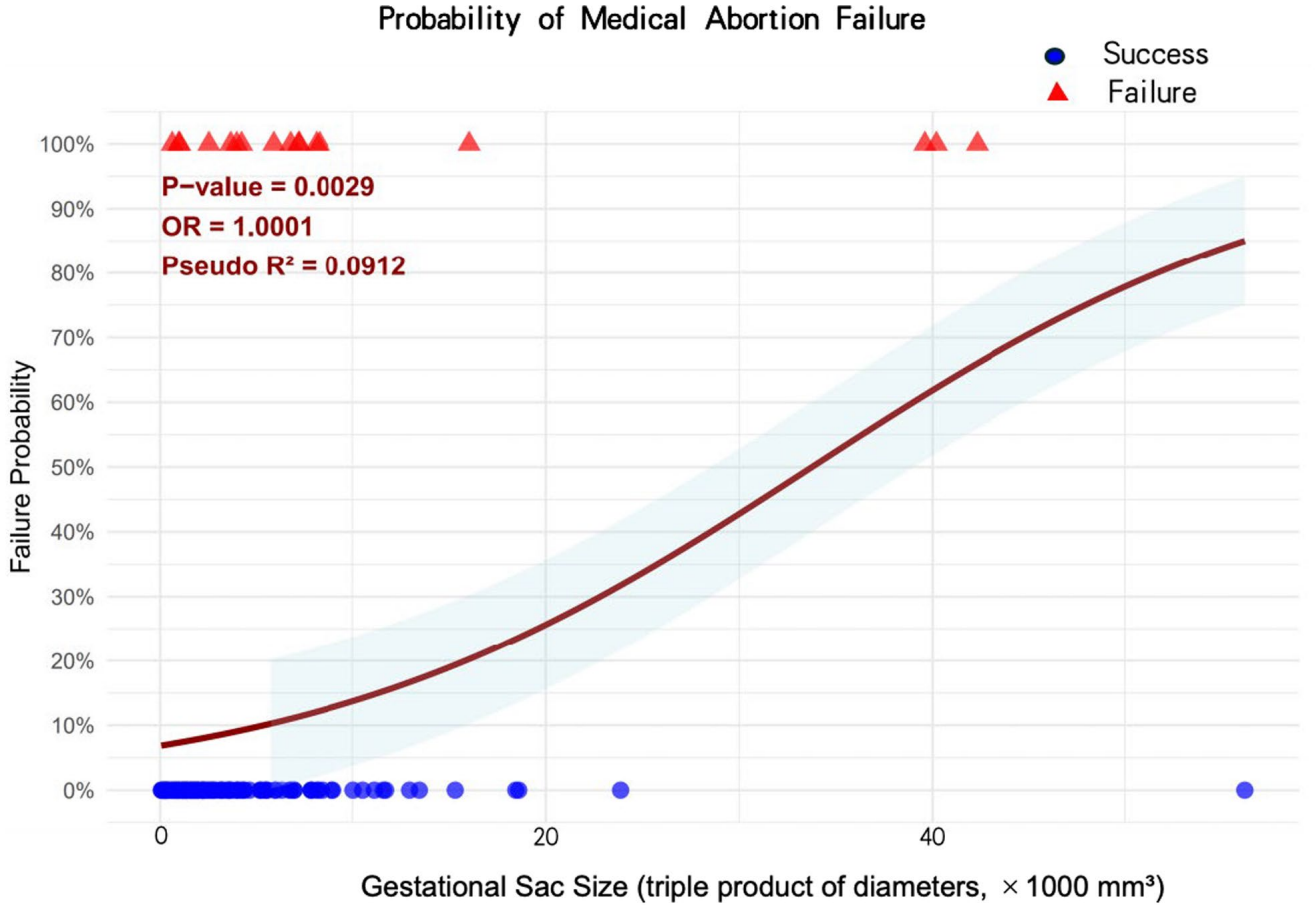
Probability of medical abortion failure by gestational sac size (triple product of diameters). This plot illustrates the association between gestational sac size (quantified as the triple product of its diameters) and the probability of medical abortion failure. Red triangles represent failed cases, while blue dots represent successful cases. The curved line denotes the predicted probability of failure, with the shaded area indicating the 95% confidence interval. Key statistical metrics include a *p*-value of 0.003, an odds ratio of 1.0001 (1.08 per 1,000 mm^3^ increase), and a Pseudo R^2^ of 0.0912, indicating a statistically significant yet modest association: As the triple product of gestational sac diameters increases, the probability of medical abortion failure rises gradually.

**Figure 5:**
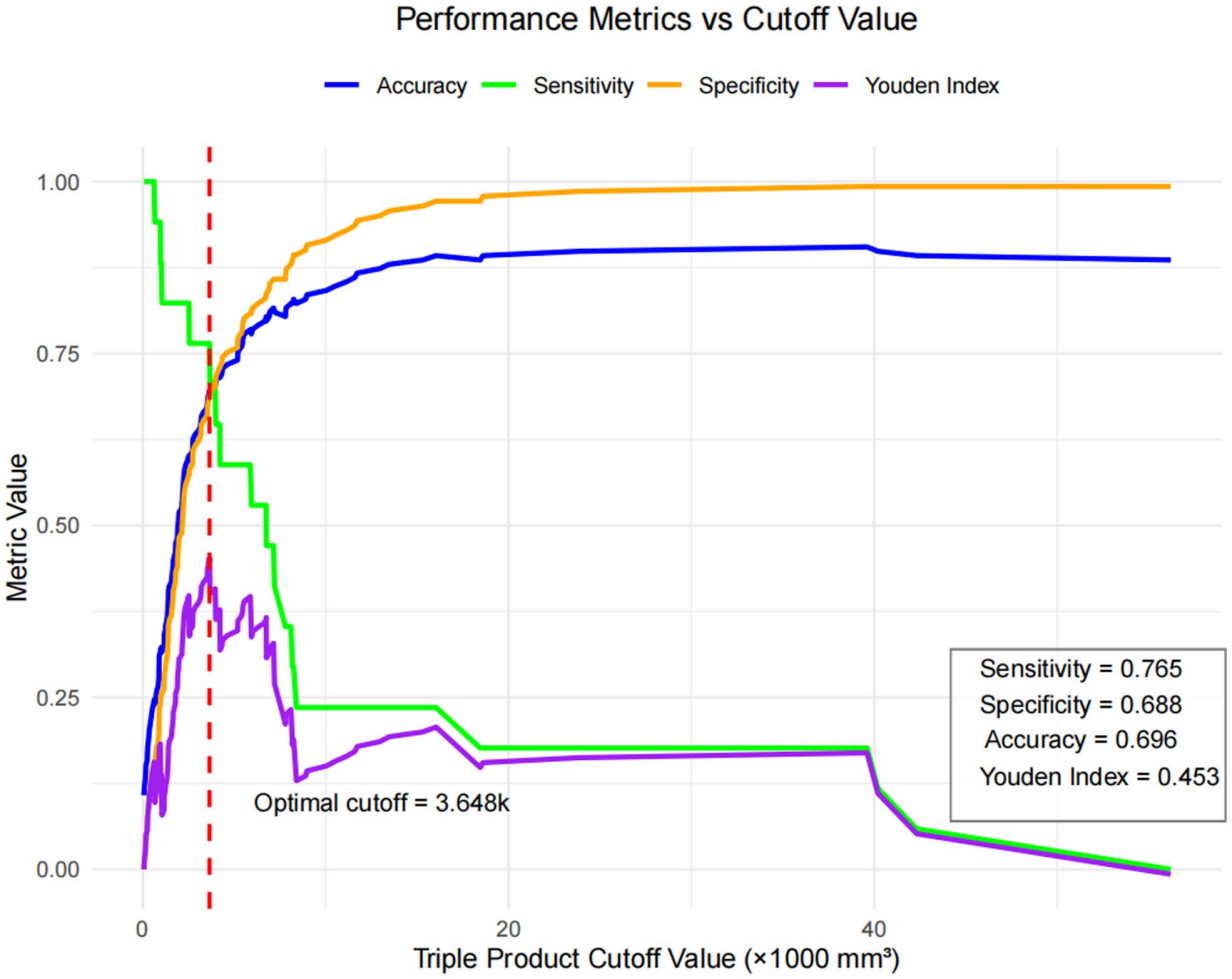
Performance metrics of the triple product cutoff value for predicting early medical abortion failure. This plot shows the accuracy (blue), sensitivity (green), specificity (orange), and Youden index (purple) across different cutoff values of the gestational sac triple product (×1,000 mm^3^). The optimal cutoff value (3,648, indicated by the red dashed line) was determined by the maximum Youden index (0.453). At this cutoff, the model achieved a sensitivity of 0.765, specificity of 0.688, and accuracy of 0.696.

For the triple product (selected for clinical measurability), analysis revealed a significant association with EMA failure with an odds ratio of 1.0001 (1.08 per 1,000 mm^3^ increase) (Figure 4). Performance metrics of the triple product cutoff value for predicting early medical abortion failure are shown in Figure 5. This plot shows the accuracy (blue), sensitivity (green), specificity (orange), and Youden index (purple) across different cutoff values of the gestational sac triple product (×1,000 mm^3^). The optimal cutoff value (3,648, indicated by the red dashed line) was determined by the maximum Youden index (0.453). At this cutoff, the model achieved a sensitivity of 0.765, specificity of 0.688, and accuracy of 0.696 (Figure 5). Supplementary Figure S1 shows a multivariable logistic regression analysis for predictors of early medical abortion failure, and gestational sac size was the strongest independent predictor of failure (OR = 10.02, 95% CI, 2.27–44.13) (Supplementary Figure S1).

### 3.5 Model performance and internal validation

The triple product model demonstrated good discrimination, with an area under the ROC curve (AUC) of 0.78 (95% CI, 0.68–0.88). After bootstrap optimism correction (1,000 resamples), the optimism-corrected AUC was 0.76 (95% CI, 0.65–0.86), indicating minimal overfitting. Calibration was assessed using the Hosmer–Lemeshow goodness-of-fit test, which showed no significant lack of fit (χ^2^ = 6.24, df = 8, *p* = 0.62). The calibration plot (see Supplementary Figure S2) displayed good agreement between predicted probabilities and observed frequencies across deciles of risk. The calibration slope was 0.94 (95% CI, 0.78–1.10) and the calibration-in-the-large was −0.05 (95% CI, −0.42 to 0.32), indicating excellent average calibration (Supplementary Figure S2).

### 3.6 Model specification

The final prediction model included only the gestational sac triple product as a predictor. The logistic regression equation was: logit(P) = −2.620 + 0.077 × (triple product/1,000), where P is the probability of early medical abortion failure, and triple product is expressed in mm^3^ (divided by 1,000 to improve interpretability of the coefficient). The odds ratio for a 1,000 mm^3^ increase in triple product was 1.08 (95% CI, 1.03–1.14; *p* = 0.003). The model’s discrimination and calibration are reported above.

## 4 Discussion

This retrospective cohort study focused on outpatients undergoing EMA with the mifepristone-misoprostol regimen, aiming to address a notable gap in EMA clinical practice: the overreliance on subjective or unvalidated metrics for failure risk assessment, such as last menstrual period (LMP)-derived gestational age (prone to bias from irregular cycles or ovulation variations) or anecdotal concerns about factors like embryonic bud presence. A key novel finding of our study—consistent with our goal of identifying high-value objective predictors—highlights that among the five gestational sac measurement indices included in this study (ellipsoid volume, maximum diameter, mean diameter, sum of three diameters, triple product), the triple product of gestational sac diameters exhibits one of the highest predictive values for EMA failure, outperforming the other indices examined (including maximum diameter and summed diameters). This finding refines our understanding of which sac size metric best translates to clinical risk stratification within the context of our study.

A central contribution of this work is twofold: first, to provide evidence to support gestational sac size as an independent predictor of EMA failure in our cohort, and second, to specify that the triple product of sac diameters is among the most robust metrics for this purpose based on our analyses. Beyond the initial association between larger sac size and higher failure risk, sensitivity analyses—adjusting for key confounders like maternal age and gestational week—confirmed that sac size (quantified via triple product) remains a standalone predictive factor for the research population. Critically, the triple product showed a stronger correlation with EMA failure (r = 0.316) compared to maximum diameter (*r* = 0.297) and summed diameters, while also demonstrating a statistically significant association with failure (OR per 1,000 mm^3^ increase = 1.08, 95% CI, 1.03–1.14, *p* = 0.003). This is clinically impactful because the triple product integrates three-dimensional sac size information (unlike single-diameter metrics), capturing a more comprehensive measure of sac volume-related growth—yet remains easy to calculate from routine ultrasound data, avoiding the complexity of ellipsoid volume formulas. This balance of comprehensiveness and practicality makes it potentially more suitable than the other studied indices for real-world clinical use in similar settings.

To further translate this insight into actionable risk stratification, we identified an optimal cutoff for the triple product (3648) via performance curve analysis, which achieved a balanced sensitivity (0.765), specificity (0.688), and Youden Index (0.453)—outperforming potential cutoffs for the other metrics included in our study. This threshold not only delivers clear guidance for clinical decision-making (e.g., flagging patients with triple product ≥3,648 for closer monitoring or additional counseling) but also aligns with our study’s goal of validating objective tools for EMA failure risk assessment in our cohort. Notably, our finding that factors like embryonic bud presence or size have no significant link to failure (all *p* > 0.05) further underscores the value of the triple product: it helps supplement vague, non-predictive concerns with a quantifiable metric, reducing unwarranted hesitation in recommending EMA for eligible patients with detectable embryonic buds. Together, these updates reinforce that the triple product of gestational sac diameters is not just one of several objective predictors examined in this study, but among the most clinically useful for EMA failure risk assessment within the context of our research.

Historically, there has been a substantial body of literature on key aspects of medical abortion, including medication administration routes, clinical settings for medication use, and the association between gestational age at medication initiation and success rates—at least based on our review of relevant studies up to the study’s initiation. However, research investigating the correlation between objective ultrasound indicators (e.g., gestational sac size and the presence or absence of an embryonic bud) and medical abortion failure remains relatively limited, particularly in the context of EMA with the mifepristone-misoprostol regimen (the focus of our current study). It was reported that those at 36 to 42 days of gestation had greater odds of success, whereas those at 50 to 56 days and 57 to 63 days had lower odds of success in comparison to the reference group (43 to 49 days) (19). Smith et al. (20) also reported a significantly higher complete abortion rate in the <56 days GA group than in the 57–63 days GA group. This was also supported by another study with a 50% higher odds of medical abortion failure in groups that had a higher proportion (>25%) of women in the ninth week of pregnancy (21). As there was no significant decrease in success rate between the 8th and 9th weeks of the GA group, this opened up possibilities of extending the same regimen up to 10 weeks of GA with not much change in success rate, which had been explored in some studies (22). Combined mifepristone-misoprostol was associated with a significantly lower rate of treatment failure, compared with misoprostol alone (23, 24). Most women prefer to undergo medical abortion at a health facility rather than at home (25), therefore, identifying the method that achieves the optimal balance between efficacy and safety for first-trimester medical abortion deserves greater attention (26). One-third of women who have a GS with cardiac activity will expel the GS with a second dose of misoprostol, making a second dose a reasonable option (27). Some important confounders were considered to have a significant impact on the occurrence of EMA failure, such as socioeconomic factors, geographic region-specific practices, and access to healthcare (3, 28, 29). Gestational age, maternal age, previous deliveries, and history of medically and surgically induced abortions all had a significant influence on the risk of EMA failure, while the inclusion of all quantified risk factors still left most interventions unpredictable (10). Furthermore, while a risk assessment model incorporating clinical parameters for predicting EMA failure has been established, gestational sac size was not included as a variable in this model (11). Ultimately, AI-driven language models have the potential to provide information on medication abortions, but there is a need for continual refinement and oversight (30).

The potential mechanisms underlying the association between larger gestational sac size and an elevated risk of medical abortion (MA) failure may include the following two aspects. First, with gestational sac enlargement, embryonic villi may undergo more extensive development, which could strengthen the area or stability of villous attachment to the uterine wall in certain cases. Second, a larger gestational sac may hinder passage through the internal cervical so in some individuals, an effect that may vary according to baseline cervical characteristics. As reported in recent studies, the success rate of MA at 9–20 weeks of gestation is lower with a 24 h interval between mifepristone and misoprostol administration than with a 48 h interval (31). We hypothesize that this difference may be related to the time-dependent cervical ripening effect of mifepristone: adequate cervical softening generally requires a sufficient duration of action, which can be modulated by factors including maternal hormone levels and baseline cervical condition. These proposed mechanisms remain theoretical, and further studies are needed to verify the above hypotheses.

Consistent with previous studies, no single parameter can perfectly predict the risk of medical abortion failure (10). At the large maternal health center in Shanghai where the authors previously worked, patients with visible embryonic buds were routinely discouraged from undergoing medical abortion due to concerns over treatment failure. However, our study found no association between the presence or size of embryonic buds and an increased risk of EMA failure in our cohort. These findings highlight the value of evidence-based practice and may reduce unnecessary hesitation in recommending EMA for eligible patients in similar settings. When choosing between medical and surgical abortion, clinicians should conduct a comprehensive risk assessment and provide thorough counseling, with the final decision fully respecting patients’ preferences.

Our study has several key strengths. First, as a cohort study conducted in a tertiary hospital affiliated with a university, it ensured relatively consistent patients’ treatment regimens, homogeneous information collection, and comprehensive variable inclusion—all of which help enhance the reliability of our conclusions within the study design. Second, to our knowledge, this is among the early studies to specifically adopt objective indicators such as gestational sac size and embryonic bud status as criteria for evaluating the risk of medical abortion (MA) failure in our target population. Compared with the subjective indicators (e.g., last menstrual period and gestational age) included in this study, these objective indicators may offer greater persuasiveness, intuitiveness, and practicality, and could be more feasible to promote in similar clinical settings. Third, data were entered manually on a case-by-case basis, followed by statistical regression analysis and sensitivity analysis. We adjusted for continuous variables (e.g., maternal age and gestational age) and categorical variables (e.g., presence of fetal heart activity and embryonic bud), which helps improve the reliability and credibility of the data for the research questions addressed.

Conversely, there are several limitations. First, it was a single-center study: while the consistency of data strengthens the conclusions, it may also introduce statistical bias and compromise the generalizability of the findings. While our study population reflects the demographics of northern Xinjiang, we acknowledge that sociodemographic factors such as socioeconomic status and healthcare access may influence model performance in other populations. However, data on these factors were not available for analysis in this study. Second, due to the retrospective nature of the study, some data obtained from outpatient medical records were missing (e.g., parity). Nevertheless, the core indicators central to our research were sufficiently comprehensive, which mitigates this limitation to some extent. Third, we recognize that baseline characteristics such as BMI and prior abortion history may influence EMA outcomes. Previous studies have reported that obesity (BMI ≥ 30 kg/m^2^) can delay fetal expulsion and increase the risk of failure by reducing misoprostol bioavailability (32, 33). Specifically, in patients with a high BMI, altered body composition leads to modified drug pharmacokinetics—primarily affecting drug distribution and clearance—and obesity has been shown to impact both drug tissue concentration and bioavailability (34). Fourth, no single ultrasound measurement of the different anatomical features in the first trimester has been shown to have a high predictive value for determining early pregnancy outcome (35); this study only compared the value of different gestational sac parameters in predicting the risk of early pregnancy medical abortion failure. Sixth, with 17 outcome events and a single predictor, the EPV was 17, exceeding the recommended threshold of 10 and indicating that overfitting is unlikely. However, due to the limited number of outcome events (EPV = 2.8), the multivariable analysis should be considered exploratory and interpreted with caution. Nevertheless, internal validation was performed to confirm model stability, and external validation in larger cohorts is still warranted. Finally, EMA failure was defined herein as the need for subsequent surgical intervention. Although we achieved complete follow-up through a combination of in-person visits and telephone interviews, the retrospective single-center design remains a limitation, and prospective multicenter studies are warranted to validate our findings.

For clinical use, the model requires three ultrasound measurements (the three perpendicular diameters of the gestational sac), which are routinely available in outpatient settings. If any of these inputs are missing or of poor quality (e.g., unclear sac margins), the model should not be applied, and clinical judgment should prevail. The model is intended for use by healthcare professionals (gynecologists, trained midwives) who can correctly obtain the measurements and interpret the predicted probabilities in the context of individual patient counseling. Before clinical implementation, external validation in diverse populations and settings is essential to assess the model’s generalizability. Future research should focus on prospective multicenter studies to evaluate the model’s performance, clinical utility, and impact on patient outcomes and shared decision-making.

## 5 Conclusion

Gestational sac size is an important objective indicator for predicting mifepristone-misoprostol EMA failure, and among various gestational sac size metrics, the triple product of diameters demonstrates the highest predictive value.

## Data Availability

The raw data supporting the conclusions of this article will be made available by the authors, without undue reservation.
