# Correction: Ultrasound-derived gestational sac triple product as a predictor of early medical abortion failure with mifepristone-misoprostol regimens: a retrospective cohort study

**DOI:** 10.3389/fmed.2026.1921012

**Published:** 2026-07-13

**Authors:** Rui-Hong Xue, Juan Li, Yun-Xia Li, Zhao-Ying Gu, Dan Tang, Jing Yang, Yan-Yan Fu, Xin-Liang Chen, Ping Liu

**Affiliations:** 1 International Peace Maternity and Child Health Hospital, School of Medicine, Shanghai Jiao Tong University, Shanghai, China; 2 Shanghai Key Laboratory of Embryo Original Diseases, Shanghai, China; 3 Institute of Birth Defects and Rare Diseases, School of Medicine, Shanghai Jiao Tong University, Shanghai, China; 4 Karamay Central Hospital, Xinjiang, China

**Keywords:** early pregnancy, failure predictor, gestational sac size, medical abortion, ultrasound

The **Ethics statement** was erroneously selected from our own files as “The study adhered to the Declaration of Helsinki, and was approved by the Human Research Ethics Committee of Karamay Central Hospital, Xinjiang, China (Review Board: YL-2025-083), which was issued on September 12, 2025. The need for informed consent to participate was waived by the Committee.”

The correct **Ethics statement** is “The study adhered to the Declaration of Helsinki, and was approved by the Human Research Ethics Committee of Karamay Central Hospital, Xinjiang, China (Review Board: YL-2025-120), which was issued on November 24, 2025. The need for informed consent to participate was waived by the Committee.”

The original version of this article has been updated.

