# Supplementary material for: Ultrasound-derived gestational sac triple product as a predictor of early medical abortion failure with mifepristone-misoprostol regimens: a retrospective cohort study
Source: Front Med (Lausanne). 2026 Apr 7;13:1780064. doi: 10.3389/fmed.2026.1780064 (PMC13096078; doi:10.3389/fmed.2026.1780064)
Supplement: Supplementary file 1 [file Data_Sheet_1.pdf]

Supplementary Materials List

Table S1 Missingness analysis of parity data

Variables	Parity Missing (n = 64)	Parity Observed (n = 95)	P value
Maternal age (y)	7 (10.9%)	35 (36.8%)	0.0003
Gestational sac size (mm)	7 (10.9%)	14 (14.7%)	0.4877
Embryonic bud (yes or no)	32 (50%)	34 (35.8%)	0.0745
Embryonic bud size (mm)	26 (40.6%)	22 (23.2%)	0.0186
Fetal heart beats (yes or no)	25 (39.1%)	27 (28.4%)	0.1607
EMA failure (yes or no)	6 (9.4%)	11 (11.6%)	0.6592

Table S1 indicates that most variables showed no significant differences. However, significant differences were observed for maternal age (P = 0.0003) and embryonic bud size (P = 0.0186). These differences indicate that the missingness of parity is associated with observed factors such as maternal age. This pattern supports the assumption that the data are missing at random (MAR).

Table S2 Sensitivity analyses of missing parity data

	Multiple Imputation (logreg)		Complete Case (CC)		Multiple Imputation (PMM)	
Variables	OR (95% CI)	P	OR (95% CI)	P	OR (95% CI)	P
Maternal age (y)	2.82 (1.00–7.95)	0.0495	3.50 (0.97–14.31)	0.0608	2.82 (1.00–7.95)	0.0495
Parity (n)	3.12 (0.41–24.01)	0.2684	3.77 (0.67–71.13)	0.2177	2.41 (0.30–19.27)	0.3979
Gestational sac size (mm)	6.40 (2.09–19.64)	0.0013	4.23 (0.97–16.85)	0.0427	6.40 (2.09–19.64)	0.0013
Embryonic bud (yes or no)	1.68 (0.61–4.64)	0.3168	2.40 (0.67–8.99)	0.1769	1.68 (0.61–4.64)	0.3168
Embryonic bud size (mm)	1.30 (0.45–3.77)	0.6289	2.10 (0.50–7.77)	0.2773	1.30 (0.45–3.77)	0.6289
Fetal heart beats (yes or no)	1.51 (0.54–4.25)	0.4343	2.35 (0.62–8.57)	0.1920	1.51 (0.54–4.25)	0.4343

Values are presented as odds ratio (95% confidence interval). P values are from pooled analyses (Rubin's rules) for MI methods and from logistic regression for CC analysis.

Abbreviations: MI, multiple imputation; CC, complete case; PMM, predictive mean matching.

Note: For variables other than Parity, MI-logreg and MI-PMM estimates are identical due to no missing data; the small difference for Parity reflects imputation algorithm variability.

Table S2 indicates that sensitivity analyses addressing missing parity data (40.3%) confirmed the robustness of our findings. After multiple imputation, gestational sac size remained the only significant predictor of abortion failure, with effect sizes consistent with the primary analysis. Complete case analysis and alternative imputation methods yielded virtually identical results.

Table S3 Multivariable analysis adjusting for all five covariates

Variables	Adjusted Odds Ratio (95% CI)	P value
Maternal age (y)	1.99 (0.59–6.68)	0.2635
Parity (n)	5.25 (0.50–55.05)	0.1634
Gestational sac size (mm)	10.02 (2.27–44.13)	**0.0026**
Embryonic bud (yes or no)	2.92 (0.32–26.57)	0.3391
Embryonic bud size (mm)	0.47 (0.08–2.92)	0.4162
Fetal heart beats (yes or no)	0.70 (0.08–6.14)	0.7474

Values are presented as adjusted odds ratio (95% confidence interval). P values are from pooled analyses using Rubin's rules across 20 multiply imputed datasets.

Abbreviations: OR, odds ratio; CI, confidence interval.

Note: Except gestational sac size, all five variables were simultaneously included in the multivariable model.

Table S3 indicates that multivariable analysis adjusting for all five covariates confirmed that gestational sac size remained the strongest independent predictor (adjusted OR = 10.02, P = 0.003).

•

Figure S1 Forest plot showing the multivariable logistic regression analysis for predictors

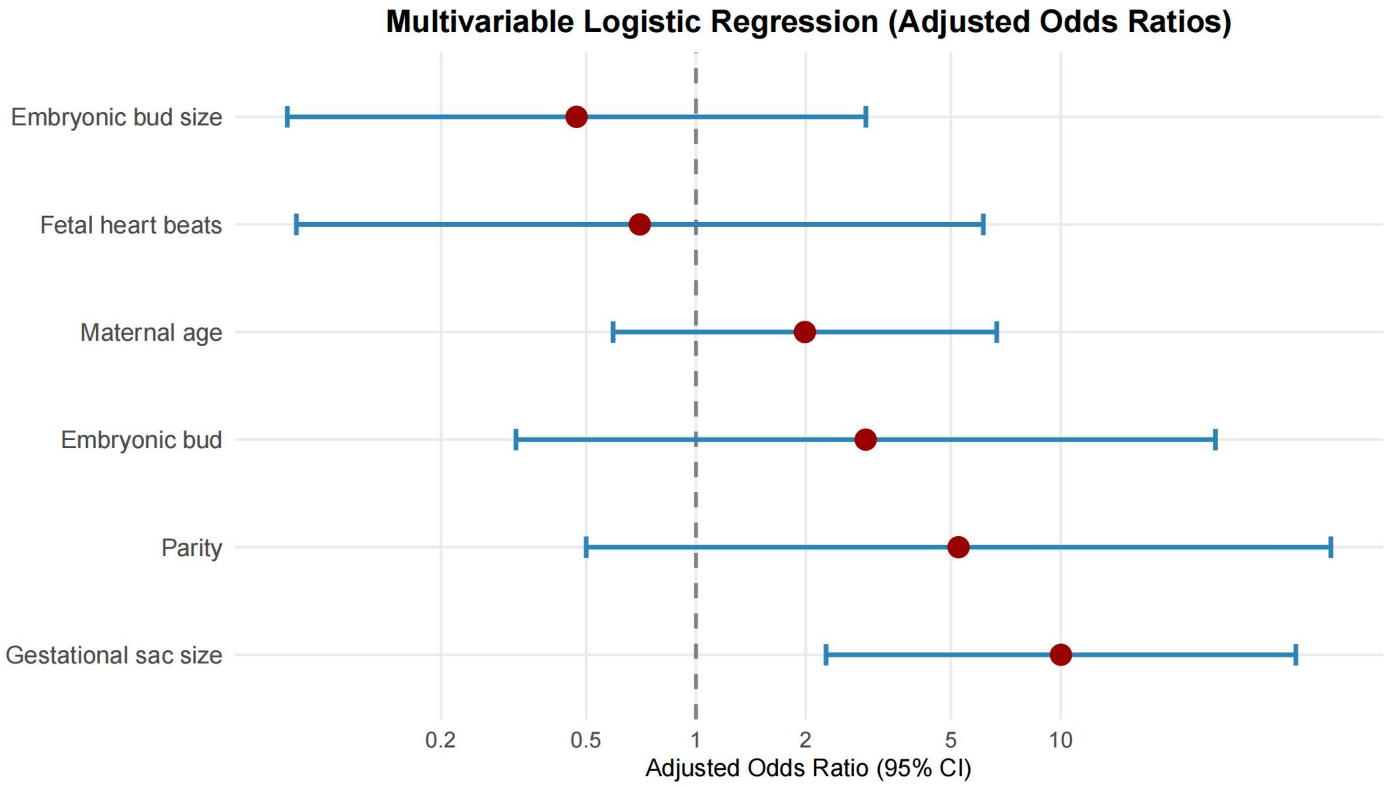

Figure S1 Multivariable logistic regression analysis for predictors of early medical abortion failure. Forest plot showing adjusted odds ratios (OR) with 95% confidence intervals (CI) for six potential predictors. Gestational sac size was the strongest independent predictor of failure (OR =

10.02, 95% CI (2.27–44.13)), while other variables including embryonic bud size, fetal heart beats, maternal age, embryonic bud presence, and parity showed no significant association. All variables were included in a single multivariable model adjusting for each other.
