# Supplementary material for: Ultrasound-derived gestational sac triple product as a predictor of early medical abortion failure with mifepristone-misoprostol regimens: a retrospective cohort study
Source: Front Med (Lausanne). 2026 Apr 7;13:1780064. doi: 10.3389/fmed.2026.1780064 (PMC13096078; doi:10.3389/fmed.2026.1780064)
Supplement: Supplementary file 2 [file Data_Sheet_2.pdf]

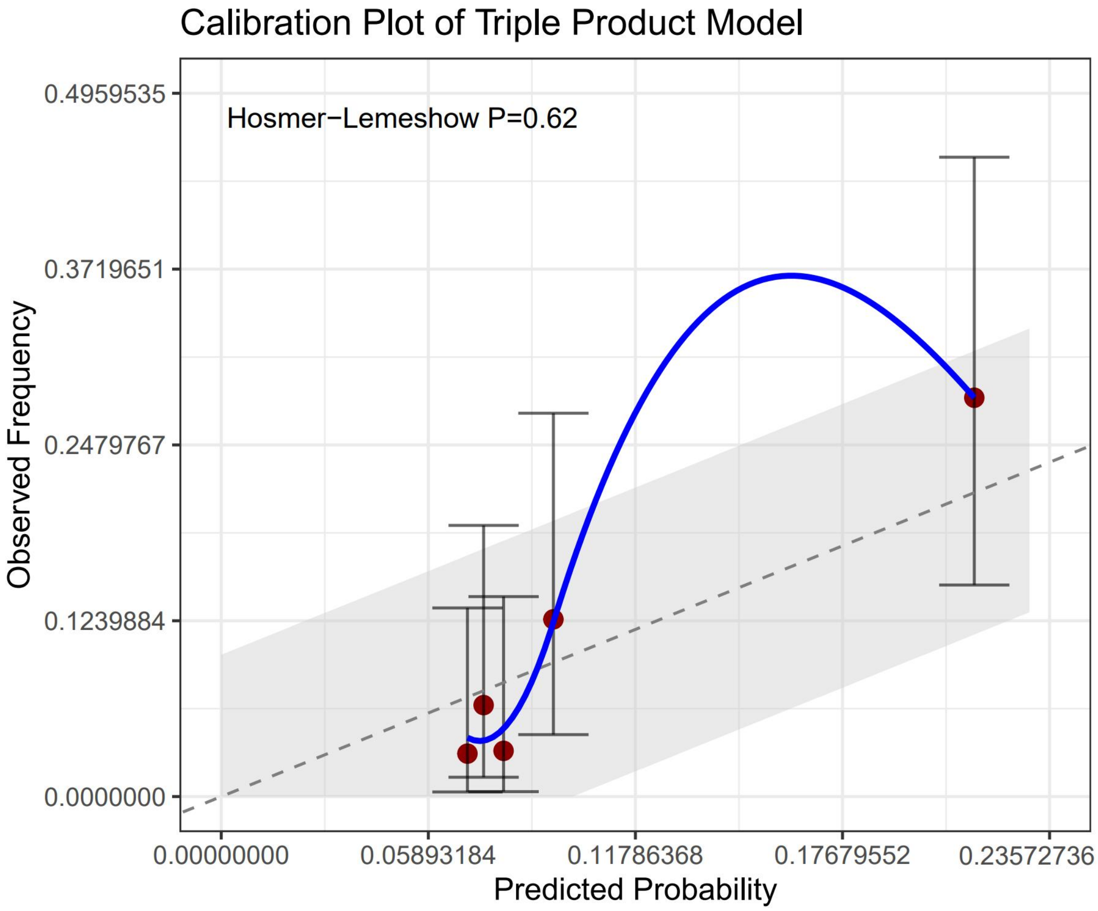

Figure S2 Calibration plot of the triple product model. The plot compares predicted probabilities (x-axis) with observed failure frequencies (y-axis). Each red point represents a risk quintile, with vertical bars showing 95% confidence intervals. The dashed diagonal line indicates perfect calibration; the gray shaded area represents ±10% deviation. The blue line is a loess smooth. Hosmer–Lemeshow P = 0.62.
